# Spatio-temporal evolution and multi-scenario simulation of land use landscape pattern in northern Guangxi

**DOI:** 10.1038/s41598-025-29312-8

**Published:** 2025-11-26

**Authors:** Tingjiang Tan, Xiangling Tang, Wei Li, Yanling Huang, Feng Xue

**Affiliations:** 1 College of Earth Sciences, Guilin University of Technology, Guilin, 541000 Guangxi China; 2 College of Tourism & Landscape Architecture, Guilin University of Technology, Guilin, 541000 Guangxi China; 3 Guilin Tourism College, Guilin, 541004 China

**Keywords:** Multi-scenario simulation, PLUS model, Landscape pattern index, Driving force mechanism, Optimisation of national land space, Ecology, Environmental sciences

## Abstract

In this study, the spatial and temporal evolution characteristics and driving mechanism of land use and landscape pattern in northern Guangxi from 1980 to 2020 were systematically analyzed by using ArcGIS 10.8, Fragstats 4.2 and PLUS models, and the land use structure and landscape pattern under different development scenarios in 2030 were simulated and predicted. The results show that: (1) Between 1980 and 2020, the areas of cropland, forest, and grassland in the northern Guangxi decreased by 307, 140, and 68 km^2^, respectively. While the area of built-up land increased by about 444 km^2^. The land use structure is characterized by ‘bidirectional succession and increased artificialization’. (2) The overall landscape pattern showed a trend of ‘increased fragmentation and decreased connectivity’, and the self-regulation ability of ecological landscape was weakened. (3) The driving analysis shows that land use change is driven by both socio-economic and natural environmental factors, among which population driving factors are dominant. (4) The results of multi-scenario simulation show that the ecological protection scenario can achieve the most compact landscape structure, the highest connectivity and aggregation, which is the optimal path for regional sustainable development. The results of this study can provide scientific basis and theoretical support for the optimal allocation of landscape space and the restoration and reconstruction of ecosystem in northern Guangxi.

**Supplementary Information:**

The online version contains supplementary material available at 10.1038/s41598-025-29312-8.

## Introduction

Landscape patterns refer to the spatial distribution and combination characteristics of landscape elements^1^, and their dynamic evolution profoundly affects ecosystem services, biodiversity, and human well-being. It has significantly changed the pattern of land use and posed a threat to ecological space and human well-being. With the acceleration of global urbanisation and the intensification of climate change, land use patterns have changed significantly, posing a threat to ecological space and human well-being^2^. The spatial and temporal heterogeneity of landscape pattern has become the core link to understand the coupling mechanism of human-land system^3^. Exploring the evolution process of landscape pattern is helpful to understand the evolution characteristics and laws of regional land use landscape, and is the basis and important support for decision makers to formulate reasonable and scientific urban planning^4^.

The current research paradigm of landscape ecology is transforming from ‘pattern-process’^5–7^ to ‘pattern-process-service-sustainability’^8–10^. The research method has gradually developed into the extensive use of 3S technology, spatial model and multidisciplinary analysis^11^. Artificial intelligence and social perception technology^12^ are promoting the innovation of analytical paradigm. From cellular automata (CA)^13^, CA-Markov^14^, CLUE-S^15^, FLUS^16^ to PLUS model^17^ which can perform high-precision spatial simulation^18^, multi-scenario prediction^19,20^, and is compatible with karst special landforms and coupled ecosystem services^21^, it has been verified in land use prediction in many regions.

The existing research has described the land use change in the western Guangxi and Beibu Gulf area^22^, and analyzed the spatial and temporal change pattern of cultivated land in Guilin City and simulated the future evolution trend^23^. These results provide a regional background for this study, but there is still a lack of unified quantitative analysis and future scenario simulation prediction for the whole region of northern Guangxi. There are limitations such as insufficient depth of regional cases^24^, unclear pattern-process coupling mechanism^25,26^, and model localization adaptation needs to be strengthened^27,28^. Therefore, this study combines ArcGIS, Fragstats and PLUS models to reveal the evolution of landscape pattern in northern Guangxi and predict future trends, aiming to solve the following key scientific issues: (1) What are the spatial and temporal evolution characteristics of land use landscape structure and pattern in northern Guangxi in the past 40 years? (2) How do natural and socio-economic factors drive the evolution of landscape pattern in northern Guangxi? (3) What is the possible evolution trend of regional landscape pattern under different development scenarios? The research can provide theoretical support and practical reference for regional ecological security guarantee and optimal allocation of land resources, and also provide a replicable technical path for land use planning and ecological security assessment in similar karst mountainous areas.

### Study area and data sources

#### Study area

**Fig. 1:**
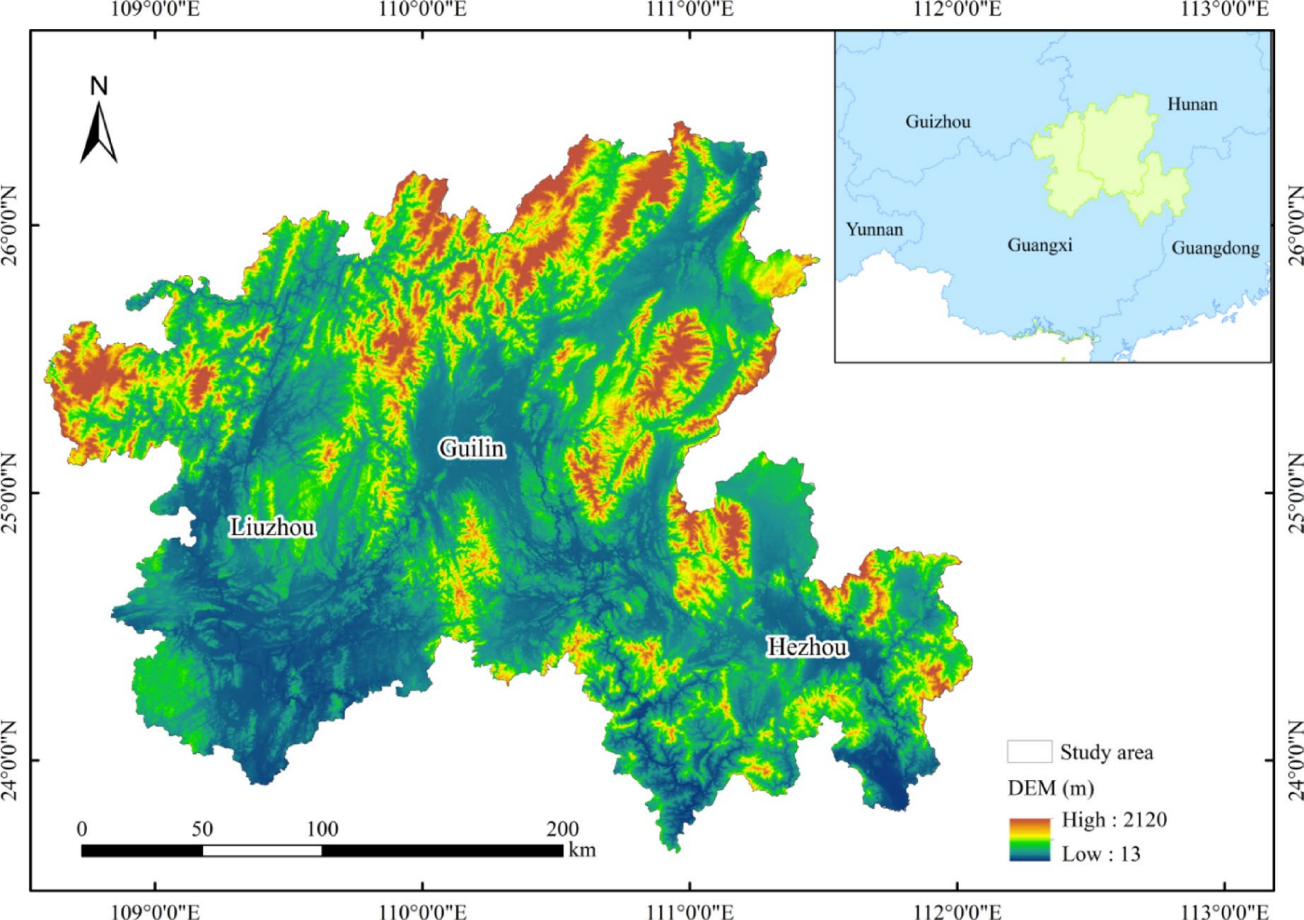
Location map of the study area. *Note* This map was created based on the standard map downloaded from the National Platform for Common GeoSpatial Information Services website (https://www.tianditu.gov.cn/(accessed on 20 November 2024), see Supplementary Materials)) with the review number GS (2024) 0650. The base map has not been modified. Same below.

The Northern Guangxi region is defined as Guilin City, Liuzhou City and Hezhou City (23°39’N-26°23’N, 109°04’E-112°03’E) in Guangxi Zhuang Autonomous Region (Fig. 1). The region is geographically connected, with similar ecological characteristics and complementary economic functions. The total area is 58,200 km^2^, which is adjacent to Guizhou Province and Hunan Province in the north and Guangdong Province in the east. The land use is dominated by forest land, cultivated land and grassland, accounting for 97% of the total area of the region, which is the background landscape of northern Guangxi. Waters, construction land and unused land accounted for less than 3%. The northern part of Guangxi is a typical karst landform with rich limestone topography. For example, ‘Guilin’s scenery is the best under heaven’ benefits from this. Its unique landscape scenery attracts a large number of domestic and foreign tourists, and plays an important role in promoting local economic and cultural exchanges. In addition, Hezhou and northern Liuzhou also have rich natural landscapes and cultural and historical sites, which together constitute an important tourism resource cluster in northern Guangxi. This unique natural landscape is not only crucial to the tourism industry, but also of great value in geological research and ecological conservation.

#### Data sources

The data used in this study include administrative division data, land use data, natural environment data and socio-economic data. The multi-period land use/land cover remote sensing monitoring database (resolution 30m) developed by the Chinese Academy of Sciences (CAS) was used for land use data, and six land types were extracted based on its first-level classification system. The database has been cross-validated by multi-period remote sensing images, and the accuracy rate is not less than 85%. The driving factor data of this study are derived from the National Platform for Common Geospatial Information Services Platform, Geospatial Data Cloud, Resource and Environmental Science Data Platform, and the National Catalogue Service For Geographic Information. The specific information is shown in Table 1. Refer to the relevant research results^17,29^, ArcGIS Desktop 10.8 (Esri, USA, https://www.esri.com/en-us/arcgis/products/arcgis-desktop/overview) was used to standardize all data to a resolution of 30m, and the coordinates were unified as WGS_1984_UTM_Zone_49N. The raster data mask was extracted to keep the spatial range consistent, and then the Euclidean distance analysis was performed on the seven accessibility factors.

**Table 1:** Data classification and sources.

Classification	Data	Data sources
Administrative division data	Guangxi provincial dataset	National Platform for Common Geospatial Information Services
Land use data	Land use classification data in Northern Guangxi from 1980 to 2020(30 m)	Multi-period land use/land cover remote sensing monitoring database (CNLUCC) developed by Chinese Academy of Sciences (CAS)
Natural environment data	Elevation (90 m)	Geospatial Data Cloud
	Slope (90 m)	Generated by elevation
	Soil type (1 km)	Resource and Environmental Science Data Platform
	The average annual temperature (1 km)	
	Annual average precipitation (1 km)	
	Distance to waters (1 km)	National Catalogue Service For Geographic Information
Socio-economic data	Population (1 km)	Resource and Environmental Science Data Platform
	GDP (1 km)	
	Distance to town center (1 km)	National Catalogue Service For Geographic Information
	Distance to rural settlements (1 km)	
	To expressway distance (1 km)	
	Distance to first-class road (1 km)	
	Distance to secondary road (1 km)	
	Distance to third-class road (1 km)	

## Methods

### Land use dynamics

Up to three levels of subheading are permitted. Subheadings should not be numbered.The dynamic degree of land use refers to the change speed of a certain type of land use within a certain time range in the region, which can quantitatively describe the change speed of land use^30^. The dynamic degree of single land use can express the quantitative change of a certain land use type in a certain period of time in a study area^31^, its equation is

1 $$K = \frac{{S_{t2} - S_{t1} }}{{S_{t1} }} \times \frac{1}{t2 - t1} \times 100\%$$

In the equation, K represents the dynamic degree of a land type in the period of t1 ~ t2; S_t1_ and S_t2_ represent the area of land use types in the early and late stages, respectively, and the fluctuation of K value reflects the number of other land types converted.

Comprehensive land use dynamic degree can express the overall land change situation^32^, its equation is

2 $$S = \left[ {\frac{{\sum\limits_{j = 1}^{n} {\Delta S_{ij} } }}{{2\sum\limits_{i = 1}^{m} {S_{i} } }}} \right] \times \frac{1}{T} \times 100\%$$

In the equation, S represents the comprehensive land use dynamic degree of a certain period of time; S_i_ is the first type of land area in the early stage of the study; ∆S_ij_ is the area from i land type to j land type; T is the duration of the study.

To comprehensively understand the intensity and direction of land use transformation in the northern Guangxi region, we selected single land use dynamics (K) and comprehensive dynamics (S) as core indicators. The K value quantifies the rate of change of specific land types, revealing the dynamic characteristics of different land use types over time. The S value reflects the intensity of aggregate changes across all land types, providing a macro-level view of the overall trends in regional land use. By combining the use of K and S, we can effectively reveal the interaction between humans and nature in the region, providing a scientific basis for subsequent analysis.

### Land use transfer matrix

The land use transfer matrix is used to reflect the mutual conversion of different land use types in the study area between the initial and final stages^33^. It can quantitatively describe the number of conversions between different land types and reveal the rate of conversion of different land use types. The equation is

3 $$S_{ij} = \left[ {\begin{array}{*{20}c} {S_{11} } & {S_{12} } & \cdots & {S_{{{1}n}} } \\ {S_{21} } & {S_{22} } & \cdots & {S_{{{2}n}} } \\ \vdots & \vdots & \vdots & \vdots \\ {S_{n1} } & {S_{n2} } & \cdots & {S_{nn} } \\ \end{array} } \right]$$

In the equation, S is the area ; n is the number of land use types before and after the transfer ; i, j (i, j = 1,2,..., n) represent the land types before and after the transfer respectively ; S_ij_ denotes the area of i-type land converted to j-type land use type before transfer.

### Landscape pattern index of land use

The landscape index can quantitatively describe the changes of landscape spatial structure and pattern. This study used ArcGIS Desktop 10.8 and Fragstats4.2, combined with the land use data of northern Guangxi from 1980 to 2020, from the two scales of patch type level and landscape level, respectively. The selection of indicators followed the following principles: (1) Clear ecological significance, directly reflecting key features of spatial configuration; (2) International applicability, facilitating cross-regional comparisons; (3) Low redundancy, avoiding duplicate calculations of highly correlated indicators; (4) Alignment with the human-land relationship issues in the study area.

In the northern Guangxi, changes in land use landscape patterns are influenced by both natural and human factors, so it is crucial to select appropriate landscape pattern indices. Table 2 lists the landscape pattern indices selected in this study and their ecological significance.

**Table 2:** Selection of land use landscape pattern indices.

Indicator	Ecological significance
NP	Directly characterising the degree of landscape fragmentation, an increase in NP value indicates that human activities have led to the fragmentation of land use patches
AREA_MN	Reflects landscape granularity and complements NP in explaining patch size distribution; smaller areas mean greater habitat fragmentation and edge effects
PAFRAC	Quantifying shape complexity and human disturbance intensity avoids the dependence of shape indices on patch size and is more suitable for landscape pattern comparisons
SHDI	SHDI calculates type richness and evenness using area weighting
SHEI	After standardisation, SHEI eliminates the influence of type numbers and, when combined with SHDI, reveals the diversity and distribution characteristics of landscape types
DIVISION	Directly measuring the spatial isolation of patches of the same type is more sensitive than simple separation formulas in responding to the fragmentation of natural habitats caused by the expansion of built-up land
PD	The number of patches per unit area comprehensively reflects the overall fragmentation trend of the landscape and eliminates the impact of regional scale differences compared to NP
LSI	Measures the complexity of landscape boundaries; higher values indicate more irregular patch shapes, unlike PAFRAC, which describes the shape of individual patches
CONTAG	Characterising landscape aggregation and connectivity, high values reflect the formation of continuous blocks of dominant types, while low values indicate a mixed distribution of multiple types
COHESION	Quantifying the physical connectivity of patches is crucial to ecological processes; it complements CONTAG, with the former focusing on spatial aggregation and the latter emphasising the actual corridor function between patches

Through comprehensive analysis of these landscape pattern indices, it is possible to fully reveal the spatio-temporal evolution characteristics of land use landscape patterns in the northern Guangxi region, providing a scientific basis for subsequent multi-scenario simulations.

### PLUS model

The PLUS model combines the rule mining framework based on the Land Expansion Analysis Strategy(LEAS) and the CA based on Multiple Random Seeds (CARS) including multiple types of random patch seeds, which can mine the driving factors of land expansion and the evolution of different scenarios^17^. Based on the land use data from 1980 to 2020, six factors (elevation, slope, soil type, annual average temperature, annual average precipitation, distance to water area) were selected from the natural environment dimension, and eight factors (population, GDP, distance to urban center, distance to rural residential area, distance to high speed, distance to first-class road, distance to second-class road and distance to third-class road) were selected from the socioeconomic dimension as predictors. The LEAS module calculates the suitability probability of each category and debugs the parameters. The CARS module is based on the transition probability. Combined with the random patches generated by automatic simulation, the parameters are set to realize the simulation of future land use landscape pattern.

Among them, the domain weight is an important index to measure the difficulty of expansion between different land use types. The value range is 0 ~ 1. The larger the value, the greater the influence of the field, the more difficult the land type is to be transformed into other land types, and the stronger the expansion ability. On the contrary, it is easier to transform into other land types. In this study, the actual situation of the transfer matrix of land use in northern Guangxi was calculated and analyzed, and the field weight value with high simulation accuracy was obtained by continuous debugging and verification in PLUS. The specific calculation equation is

4 $$X^{*} = \frac{{X - X_{{{\mathrm{min}}}} }}{{X_{{{\mathrm{max}}}} { - }X_{{{\mathrm{min}}}} }}$$

In the equation, X* represents the standardized deviation value, X is the change area of each land use type between the two periods of land use data ; X_max_ is the maximum value of the area change of all land types, and X_min_ is the minimum value of the area change of all land types. The domain weights of cropland, forest land, grassland, water area, construction land and unused land are 0.915164, 0.124816, 0.527784, 0.309434, 0.99 and 0.1 respectively.

^*^Note : * According to the actual expansion of land, the land use type with a weight of 0 is artificially set to 0.1, and the land use type with a weight of 1 is artificially set to 0.99.

Referring to the research results of relevant scholars^16,34–37^, and according to the actual situation of land use in northern Guangxi, the future land use spatial change in northern Guangxi is set to four scenarios : natural development scenario, urban development scenario, cropland protection scenario and ecological protection scenario. Adjust the land use transfer probability in the Markov chain, and obtain the area of each land use type under each scenario. The specific settings are as follows: (1) Natural development scenario: Continuing the development trend from 1980 to 2020, without limiting the expansion of construction land, to ensure the objectivity of the prediction results. (2) Urban development scenario: Combined with the ‘Guangxi New Urbanization Plan (2021–2035)’, considering that after China has built a well-off society in an all-round way, people’s demand for a better life is increasing, and urban development is further accelerated. (3) Cropland protection scenario: combined with the requirements of ‘Guangxi Zhuang Autonomous Region Land Spatial Planning (2021–2035)’, implement the most stringent cultivated land protection system to ensure food security. Through the superposition analysis of the cropland data from 1980 to 2010, the areas with cropland in four years were extracted as the long-term stable cropland in northern Guangxi. (4) Ecological protection scenario: Based on the concept of ‘lucid waters and lush mountains are invaluable assets’, combined with the actual land use situation in northern Guangxi, the forest land, grassland and water area are defined as ecological land, and the water area is set as a restricted transfer area to reduce the transfer of ecological land to other land types, while limiting the transfer of cultivated land and construction land to ecological land.

The land use transfer matrix under different scenarios is shown in Table 3, where ‘1’ indicates that the land type can be transferred to other land types, and ‘0’ indicates that the land type cannot be transferred to other land types.

**Table 3:** Transfer cost matrix under different scenarios. A ~ F correspond to cropland, forest, grassland, water, built-up land and unused land.

2020–2030	Natural development scenario						Urban Development Scenario					
	A	B	C	D	E	F	A	B	C	D	E	F
A	1	1	1	1	1	1	1	0	0	0	1	1
B	1	1	1	1	1	1	1	1	1	0	1	1
C	1	1	1	1	1	1	1	1	1	1	1	1
D	0	0	0	1	0	0	0	0	0	1	1	0
E	0	0	0	0	1	0	0	0	0	0	1	0
F	1	1	1	1	1	1	1	1	1	1	1	1

2020–2030	Cropland protection scenario						Ecological protection scenario					
	A	B	C	D	E	F	A	B	C	D	E	F
A	1	0	0	0	0	0	1	1	1	1	1	1
B	1	1	1	0	1	1	0	1	0	0	0	0
C	1	1	1	1	1	1	0	1	1	0	0	0
D	1	0	0	1	0	0	0	1	1	1	0	0
E	0	0	0	0	1	0	0	0	0	0	1	0
F	1	1	1	1	1	1	1	1	1	1	1	1

## Results

### Analysis of land use change

**Fig. 2:**
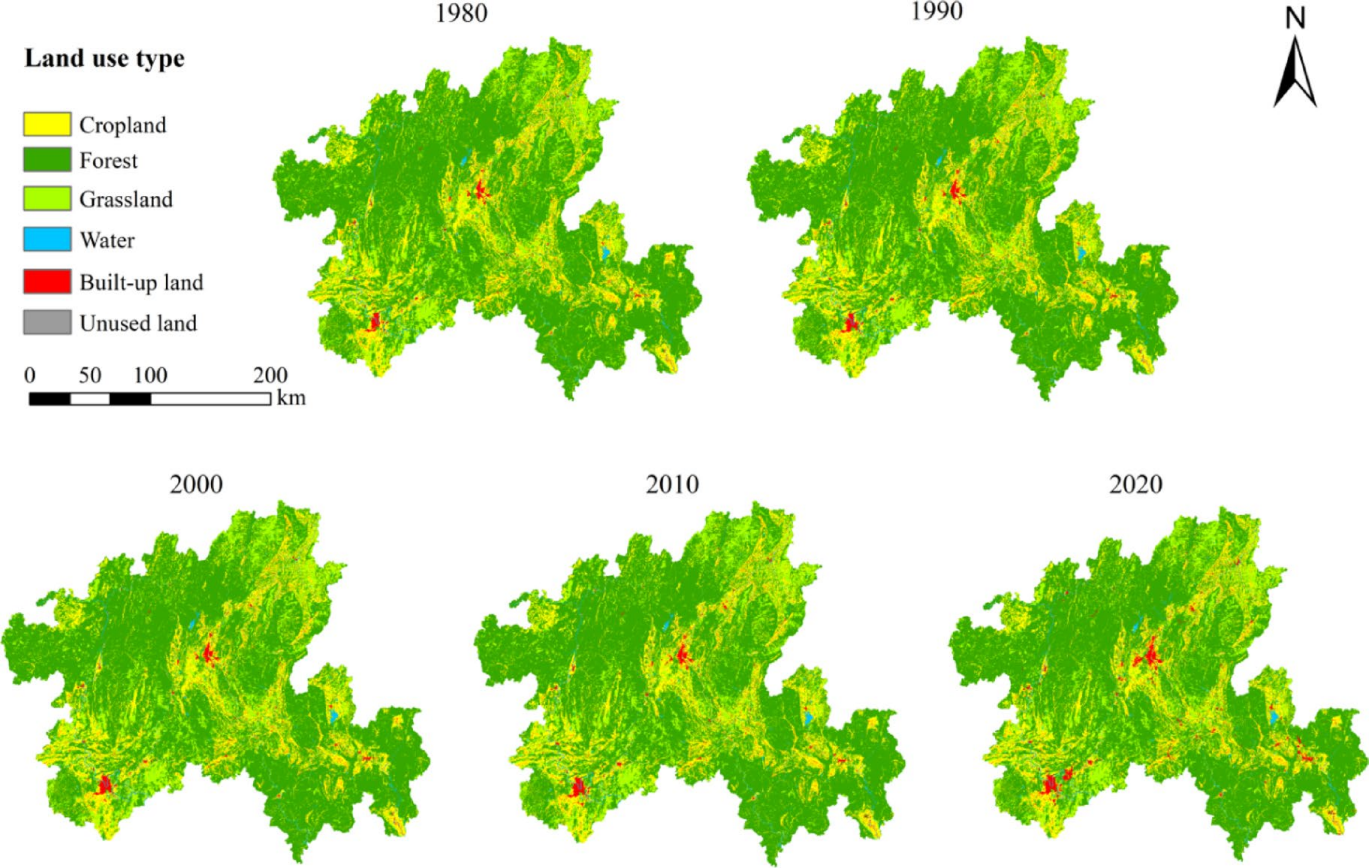
Spatial distribution map of land types in northern Guangxi from 1980 to 2020.

From the perspective of the dynamic changes of land use types (Fig. 2 & Table 4), in the past 40 years, the background landscape advantages of cropland, forest and grassland have continued to decline, and the areas have decreased by 307 km^2^,140 km^2^ and 68 km^2^, respectively. The decline in landscape advantage confirms the erosion of natural landscapes by human activities. On the contrary, the area of water, built-up land and unused land increased by 67 km^2^, 444 km^2^ and 2 km^2^ respectively. Among them, the built-up land is radially distributed, with the largest increase of 4.86%, the rapid expansion of built-up land is a direct reflection of regional urbanisation. The area of unused land was small, only increased by 2 km^2^, and all converted to other land types in the later stage of the study. From 1980 to 2010, the comprehensive dynamic degree of land use was stable between 0.01 and 0.02, while from 2010 to 2020, it rose sharply to 0.06, revealing a sharp increase in human intervention over the past decade, landscape transformation has entered an accelerated phase. According to the transfer matrix of land use change in northern Guangxi from 1980 to 2020 (Table 5), the transferred area is cropland > forest > grassland > built-up land > water > unused land, and the transferred area is built-up land > forest > grassland > cropland > water > unused land. Among these, although cropland has natural base attributes, it is essentially an artificial natural and artificially managed landscape formed by long-term human cultivation activities (Current land use classification GB/T 21010-2007)^38^. Cropland has mainly been converted into built-up land and forest. The conversion of cropland into built-up land reflects the transition from semi-naturalised human-made landscapes to highly intensified human-made landscapes. Another portion of cropland has been converted into forest, while the mutual conversion between forest and grassland reflects the process of natural restoration in the northern Guangxi. This process demonstrates the bidirectional nature of landscape succession, collectively confirming the significant increase in the degree of landscape artificialization under human activity.

**Table 4:** Land use dynamic change in northern Guangxi from 1980 to 2020.

Time period	Statistical type	A	B	C	D	E	F	Comprehensive dynamic degree/%
1980–1990	Net variable/km^2^	−35.75	−69.07	69.25	0.72	34.60	0.00	0.02
	Dynamic attitude/%	−0.03	−0.02	0.10	0.01	0.42	0.00	
1990–2000	Net variable/km^2^	−23.85	35.17	−39.41	3.18	25.00	0.00	0.01
	Dynamic attitude/%	−0.02	0.01	−0.06	0.05	0.29	0.00	
2000–2010	Net variable/km^2^	−48.17	48.70	−92.00	32.60	58.10	1.08	0.02
	Dynamic attitude/%	−0.04	0.01	−0.13	0.48	0.66	4.11	
2010–2020	Net variable/km^2^	−199.03	−154.55	−6.07	30.35	326.65	1.14	0.06
	Dynamic attitude/%	−0.18	−0.04	−0.01	0.42	3.49	3.07	
Grand total	Net variable/km^2^	−307	−140	−68	67	444	2	–

A ~ F correspond to cropland, forest, grassland, water, built-up land and unused land.

**Table 5:** Land use dynamic change in northern Guangxi from 1980 to 2020.

1980	2020						Unit: km^2^
	Cropland	Forest	Grassland	Water	Built-up land	Unused land	Roll-out
Cropland	10,414.69	114.13	23.14	40.83	292.51	0.77	471.39
Forest	115.75	38,372.67	189.46	26.37	116.59	1.46	449.63
Grassland	25.15	186.85	6508.13	16.03	55.77	0.01	283.81
Water	6.98	5.83	2.23	661.58	3.01	0.01	18.06
Built-up land	16.66	3.73	1.43	1.72	794.45	0.00	23.54
Unused land	0.00	0.03	0.01	0.00	0.00	2.58	0.04
Roll in	164.54	310.57	216.27	84.95	467.88	2.25	–

### Landscape pattern change analysis

#### Type level analysis

**Fig. 3:**
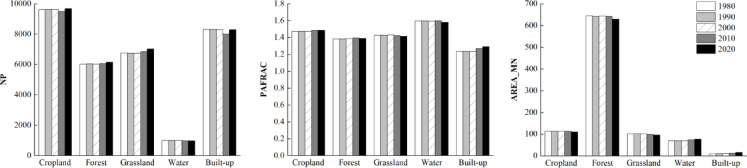
Landscape index and change of land use types in north Guangxi from 1980 to 2020.

At the patch type level (Fig. 3), the number of patches (NP) of each land use type fluctuated from 1980 to 2020, among which cropland, forest and grassland still dominated. From the perspective of ecological process, the decrease of average patch area (AREA_MN ) indicates that the landscape of cropland, forest and grassland is gradually fragmented, forming more small patches, which directly weakens the continuity of habitat, hinders species migration, and has a negative impact on biodiversity. The average patch area of water and built-up land continued to rise, showing an intensive trend, resulting in further compression of the surrounding natural habitat and increasing the degree of fragmentation. The perimeter-area fractal dimension (PAFRAC) shows that the patch boundaries of cropland, grassland and water are irregular and fragmented, which are greatly disturbed by human activities. The patch shape of forest and built-up land tends to be simple. At the level of ecological process, irregular boundaries aggravate the edge effect, change the edge environment, and then affect the composition and function of plant communities.

#### Landscape level analysis

It can be seen from Table 6 that the patch density (PD) and landscape shape index (LSI) showed an increasing trend at the landscape level in northern Guangxi from 1980 to 2020. The increase of patch density (PD) indicates that the degree of landscape fragmentation is aggravated, and more land is divided into independent small patches, which may be closely related to land use changes such as urbanization, agricultural expansion and road construction. The increase of landscape shape index (LSI) reflects the complexity of the shape of landscape units (patches), indicating that the landscape structure tends to be irregular and the boundary is blurred, which further aggravates the degree of landscape fragmentation.

**Table 6:** Landscape index of different periods in Northern Guangxi.

Year	PD	LSI	CONTAG	COHESION	DIVSION	SHDI	SHEI
1980	0.5463	135.7796	68.8997	99.8923	0.9181	0.9465	0.5283
1990	0.5466	136.1347	68.7857	99.892	0.9185	0.9501	0.5303
2000	0.5462	136.0225	68.784	99.8927	0.9179	0.9503	0.5304
2010	0.5406	137.6236	68.667	99.8914	0.9184	0.9527	0.5317
2020	0.5539	139.916	68.0832	99.8899	0.9195	0.9704	0.5416

The decrease of contagion index (CONTAG) indicated that the connectivity between patches was weakened, the landscape expansion ability was reduced, and the transition between different land use types tended to be dispersed and the continuity was insufficient. The decline of landscape connectivity index (COHESION) reflects the decrease of spatial connectivity of patches within the landscape, the fragmentation of ecological networks, the decline of ecosystem resilience, the limitation of ecological diversity, and the decline of regulatory service capacity. The increase of (DIVISION) indicates that the degree of isolation between patches is increased, which leads to the decrease of interoperability and connectivity of biological communities, which in turn has a negative impact on the stability and resilience of ecosystems.

The Shannon’s diversity index (SHDI) and Shannon’s evenness index (SHEI) showed an upward trend, indicating that the distribution of land use types was more abundant, and the spatial distribution of different types of patches tended to be balanced. Although it improves landscape heterogeneity, it is still difficult to translate into ecological benefits if there is a lack of functional connectivity.

### Analysis of the driving forces behind changes in land use types

**Fig. 4:**
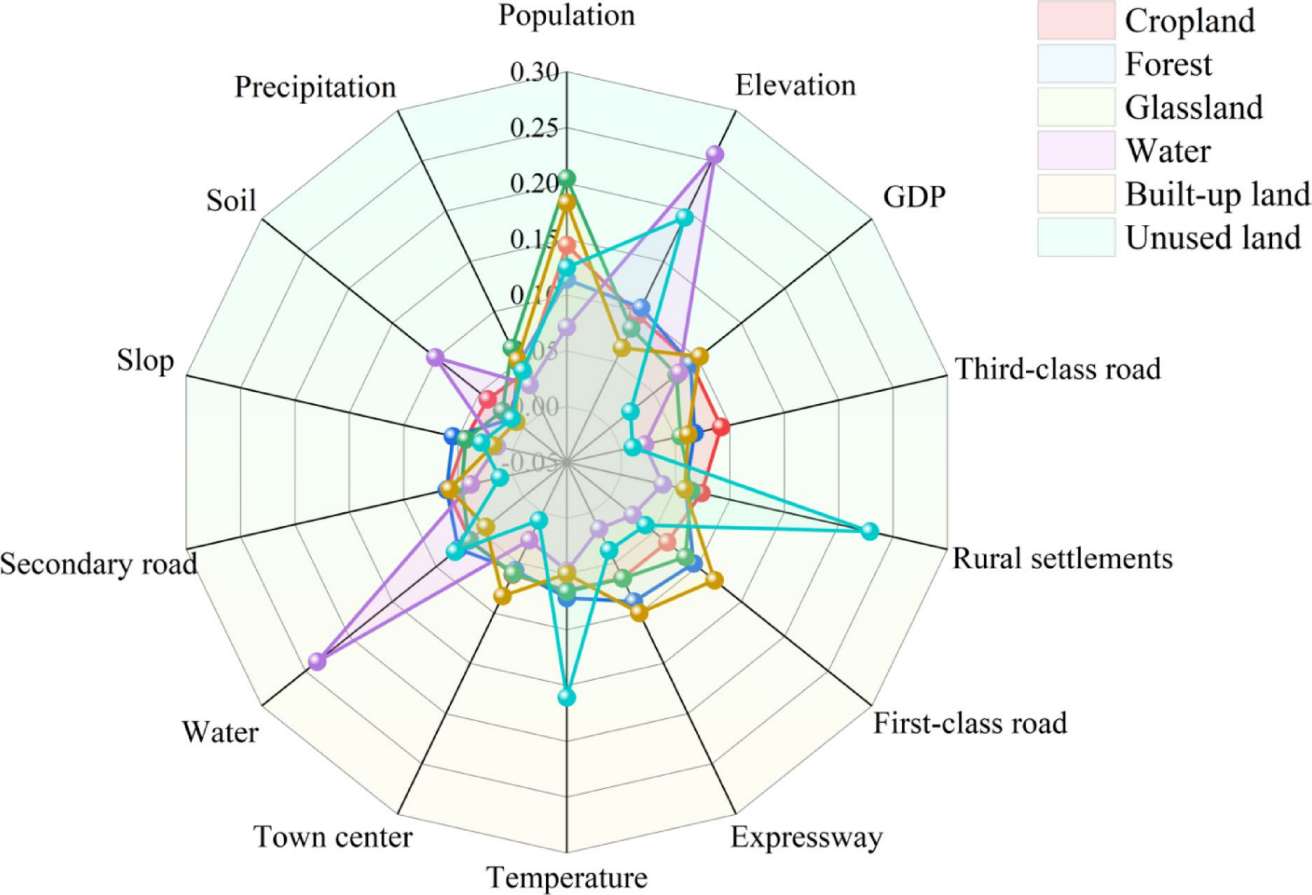
Contribution degree of each land use expansion driver.

**Fig. 5:**
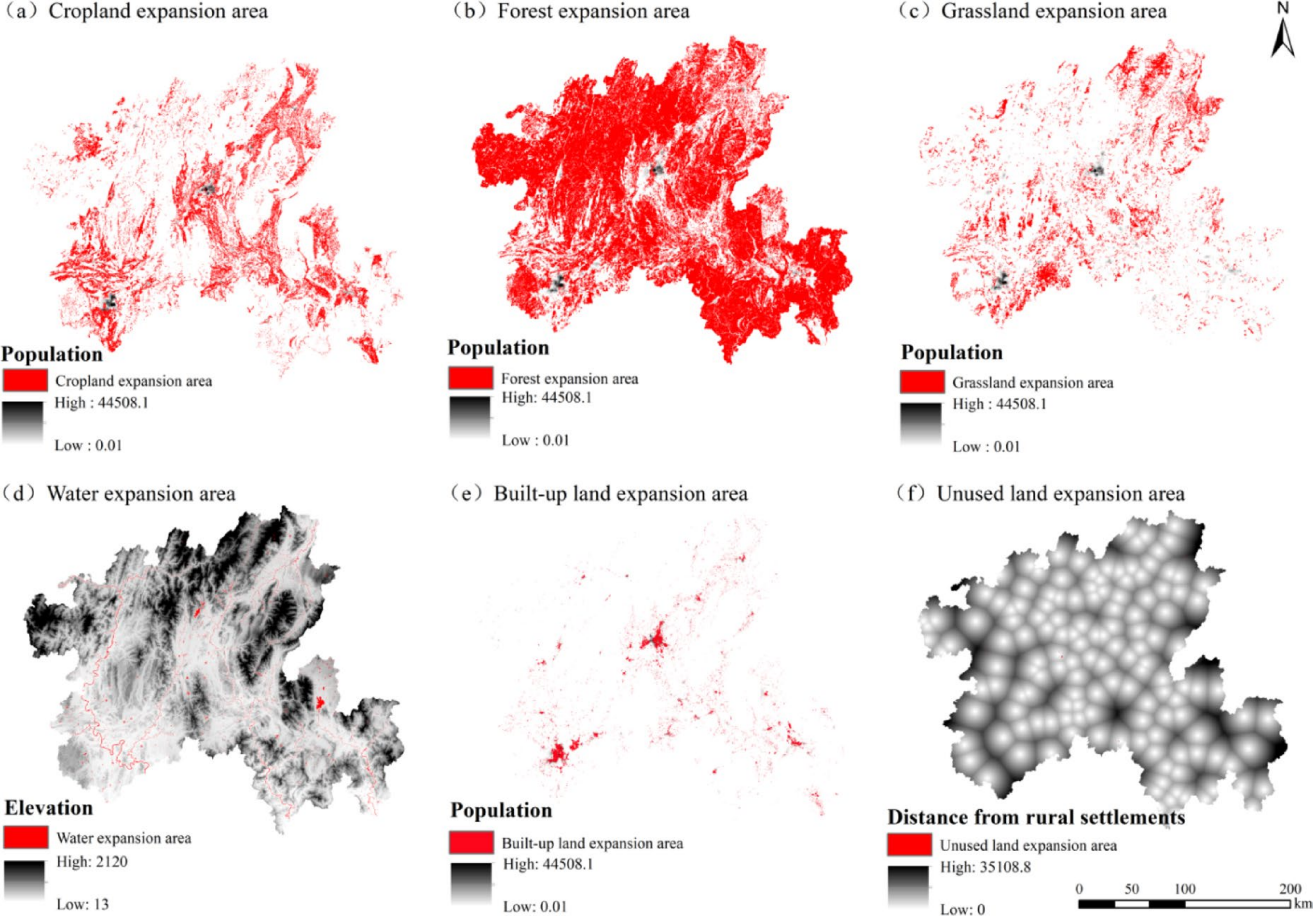
Overlay map of expansion areas by location and corresponding maximum contribution factors.

This study used land use data from 1980 and 2020 and the Land Expansion Analysis Strategy (LEAS) module in the PLUS model to simulate and calculate the contribution of driving factors to changes in land use types in the northern Guangxi (Fig. 4). The expansion areas of each land type were then superimposed on the corresponding highest contributing factors (Fig. 5) to analyse the driving forces behind changes in each land type.

As shown in Figs. 4 and 5, cropland expansion is driven by population growth and GDP. The areas of cropland expansion highly overlap with regions of high population density, and expands to the surrounding areas with three core urban areas. Indicating that population growth and increased demand for food are the primary drivers of cropland expansion. Population-dense areas have a higher demand for cropland to meet both food production and living needs. At the same time, elevation constrains the distribution of cropland, and low-altitude, flat terrain areas provide suitable farming conditions.

The expansion of forest mostly occurs in sparsely populated mountainous areas and high-altitude areas. The expansion area of forest is highly overlapped with the low population density area, which is significantly affected by human interference, with population expansion and road construction driving the reduction of forest, revealing that human activities are the primary cause of forest degradation. The reduction of human activities is conducive to the restoration or expansion of forest land.

Grassland expansion is wide and scattered, mainly distributed in low population density, moderate slope or medium and high altitude areas. Population density is still the main factor, but its effect is relatively weaker than that of cropland and built-up land, reflecting the compound effect of grassland reclamation, ecological restoration and natural vegetation succession.

The expansion of waters is concentrated in low-altitude areas, which is in line with the terrain conditions naturally formed by water bodies and strongly depends on natural geographical factors. Elevation is a key natural factor determining the distribution of waters, and social factors such as population have less influence.

The expansion area of built-up land is closely superimposed on the population density area, showing the spatial characteristics of the urbanization process. Socio-economic factors such as population density, GDP, urban center of gravity and road network are highly correlated and are the core engines of urban expansion.

Unused land is highly correlated with distance from rural settlements, with areas farther from rural settlements more likely to revert to unused land, primarily through the conversion of abandoned homesteads and fallow land.

In summary, the land use change in northern Guangxi is the result of the interaction of socio-economic and natural environmental factors, and the population is the main driving factor. Population density and economic activities are the main driving forces for the expansion of cultivated land and construction land. Natural factors such as elevation dominate the distribution of water areas ; the changes of forest land and unused land are more negatively affected by the intensity of human activities. The above driving factor relationship and spatial distribution are intuitively verified in Figs. 4 and 5, which provides a clear basis for subsequent land use planning.

### Future scenario simulation analysis

#### Simulation accuracy analysis

**Fig. 6:**
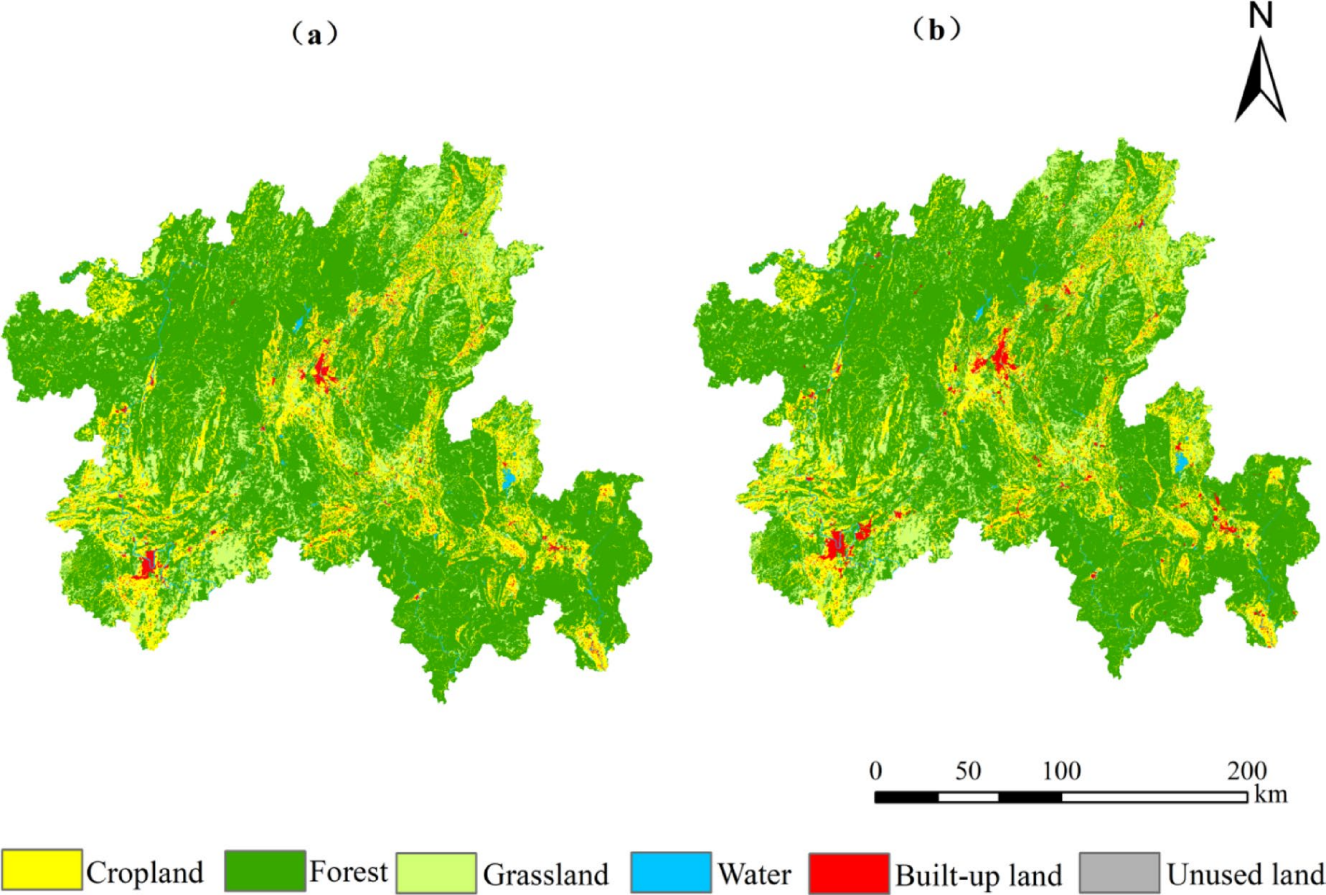
PLUS land use simulation results and real land use status comparison map. (**a**) Land use simulation results in 2020. (**b**) Actual land use status in 2020.

Based on the PLUS model and the selected 14 driving factors, the land use data of Northern Guangxi from 1980 to 2010 were used as the training set to predict the spatial distribution of land use in 2020 (Fig. 6). In order to further verify the simulation accuracy, the Kappa coefficient is 0.94, which meets the ideal requirements of simulation and can be used for the prediction and analysis of land use in Northern Guangxi. Because land use change is affected by many factors, the change process is more complex, which leads to many possibilities of future land use spatial pattern change in Northern Guangxi.

#### Land use change under different scenarios in northern Guangxi in 2030

**Fig. 7:**
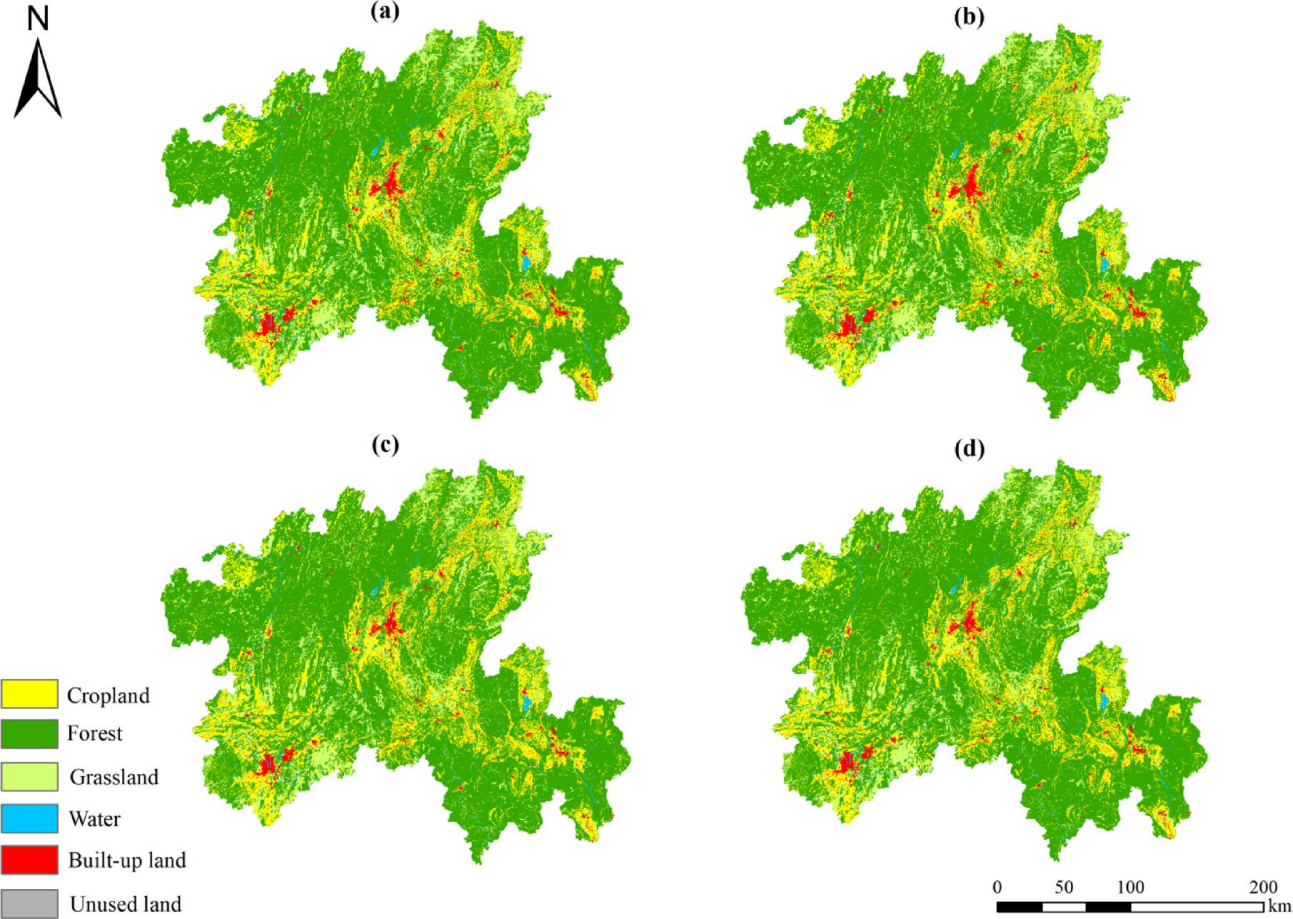
Simulation of scenarios for northern Guangxi in 2030. (**a**) Natural development scenario. (**b**) Urban development scenario. (**c**) Cropland protection scenario. (**d**) Ecological protection scenario.

According to the simulation results of land use pattern in northern Guangxi under four scenarios in 2030 (Fig. 7 & Table 7), there are obvious differences in land use changes under different scenarios, which will have different effects on the development of the study area. Compared with 2020, under the natural development scenario, the cropland, forest and grassland decreased by about 206,488 km^2^,170,278 km^2^ and 6481 km^2^, respectively. The area of water area and built-up land increased steadily and slowly, increasing by about 32,845 km^2^and 350,457 km^2^, respectively. The area of unused land was small and temporarily neglected. All land use types develop freely, and the direction of change is consistent with previous years, but the significant reduction of agricultural and ecological land needs attention.

**Table 7:** Land demand in different scenarios.

Type	Cropland	Forest	Grassland	Water	Built-up land	Unused land
2030 Natural development scenario	11,541,437	42,787,185	7,460,831	861,865	1,752,208	5319
2030 Urban development scenario	11,749,310	42,304,705	7,627,256	811,291	1,911,664	4619
2030 Cropland protection scenario	11,920,957	42,788,621	7,461,141	765,925	1,467,086	5115
2030 Ecological protection scenario	11,604,042	43,035,138	7,485,928	781,328	1,498,028	4381

Under the scenario of urban development, the area of forest and water decreased by about 652,758 km^2^ and 17,729 km^2^ respectively, and the built-up land increased by nearly 36%, about 509,913 km^2^. In this scenario, the northern Guangxi is dominated by rapid economic and social development, and the urban area of northern Guangxi is expanding rapidly outward, ignoring ecological governance and protection, threatening agricultural land and ecological land, which is not conducive to urban development in the long run. It may aggravate the contradiction between people and land and affect regional ecological security.

Under the scenario of cropland protection, forest is still the main land use type, followed by cropland, while other land use types account for a relatively small share. The cropland and built-up land are developing positively, and the forest, grassland and water are slightly decreased, about 168,842 km^2^, 6171 km^2^ and 63,095 km^2^, respectively. The northern Guangxi region is relatively stable. It is the middle path of both development and protection.

Under the ecological protection scenario, the area of forest and grassland was restored, which increased by 77,675 km^2^ and 18,616 km^2^ respectively, and the area of cropland and water decreased by about 143,883 km^2^ and 47,692 km^2^ respectively. The forest and grassland with high ecological value have been protected to a certain extent. The occupation of surrounding cropland by built-up land has slowed down, the ecosystem has been improved to a certain extent, and the ecological benefits have increased significantly. This is consistent with the strategic orientation of enhancing the ecological barrier function in the main functional area plan of Guangxi, and is conducive to the long-term sustainable development of the region.

#### Analysis of landscape pattern under different scenarios in northern Guangxi in 2030

**Fig. 8:**
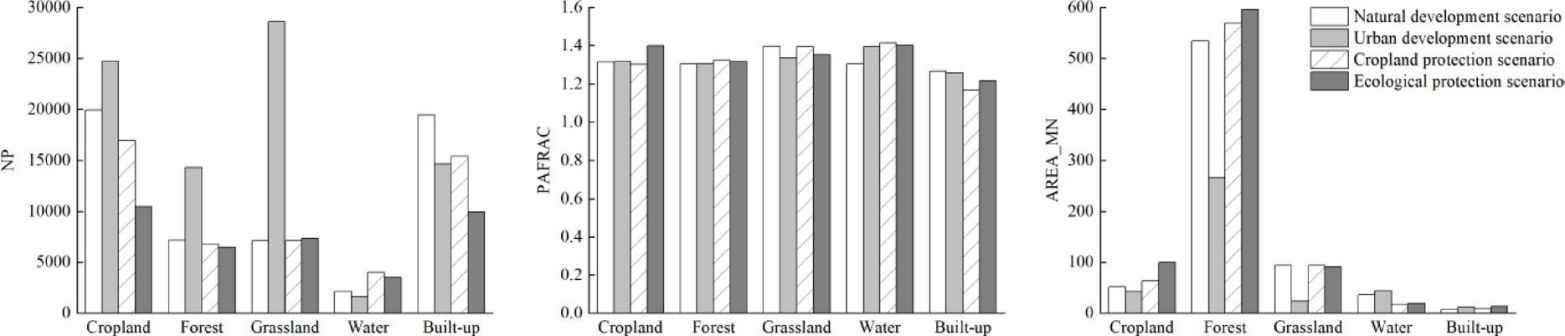
Landscape level index and changes of land use types under different scenarios in northern Guangxi in 2030.

**Fig. 9:**
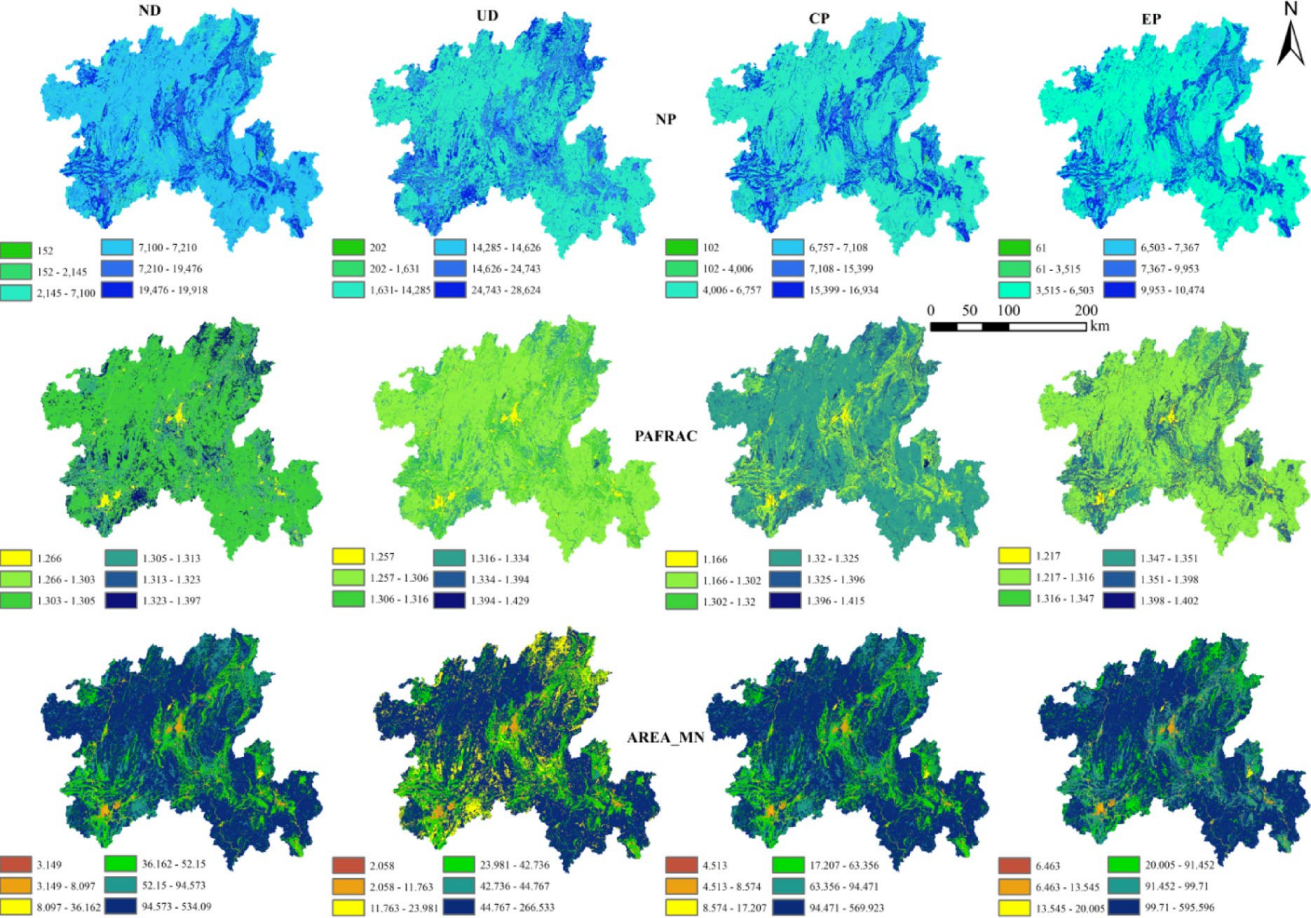
The evolution of land use type level under different scenarios in northern Guangxi in 2030.

Based on the land use data under each scenario in 2030, Fragstats4.2 software was used to calculate the landscape pattern index under each scenario in 2030 and analyze the landscape pattern. The results are shown in Fig. 8, Table 8 and Fig. 9.

**Table 8:** Landscape index of different scenarios in north Guangxi.

Type	PD	LSI	CONTAG	COHESION	DIVSION	SHDI	SHEI
2030 Natural development scenario	0.9661	160.8417	67.0472	99.8873	0.9192	0.986	0.5503
2030 Urban development scenario	1.451	184.4835	66.0531	99.8894	0.9207	0.9993	0.5577
2030 Cropland protection scenario	0.8678	153.2943	67.6133	99.8942	0.9187	0.9732	0.5432
2030 Ecological protection scenario	0.6533	148.7403	67.8464	99.9425	0.7299	0.97	0.5414

At the patch type level, the four scenarios showed consistency: the number of patches (NP) increased, the average patch area (AREA _ MN) and the perimeter-area fractal dimension (PAFRAC) decreased. This shows that in 2030, the number of land use types will increase, the degree of landscape fragmentation will increase, the shape of patches will be more complex, and the interference of human activities on the landscape will be further enhanced.

At the landscape level, in the natural development scenario in 2030, the patch density (PD) and landscape shape index (LSI) increased compared with 2020, while the contagion index (CONTAG) and landscape connectivity index COHESION) decreased slightly. The landscape isolation (DIVISION), diversity index (SHDI) and evenness index (SHEI) remained stable. This shows that the landscape fragmentation in northern Guangxi shows a uniform growth trend, and the patch shape is more fragmented, but the distribution and diversity of landscape types remain relatively stable.

In the 2030 urban development scenario, the patch density (PD), landscape shape index (LSI) and landscape separation index (DIVISION) increased significantly, it reflects the increase of landscape fragmentation and shape complexity. While the contagion index (CONTAG) and landscape connectivity index (COHESION) decreased slightly. It shows that the overall aggregation degree decreases slightly, but the connectivity remains at a high level. The diversity index (SHDI) and evenness index (SHEI) increased slightly. It shows that the distribution of different land types is more uniform. The urban development scenario leads to a sharp increase in the number of patches, a reduction in the area, and an increase in the degree of landscape fragmentation. The interference of human activities on landscape connectivity and ecological processes is intensified, the ability of landscape to resist risks is significantly reduced, and the landscape security is at the lowest level in the four scenarios. Although the overall connectivity is still high, the ecological function may be negatively affected by fine patches, and the planning of ecological corridors and green space networks needs to be strengthened.

In the scenario of cropland protection in 2030, the patch density (PD), landscape shape index (LSI), landscape connectivity index (COHESION), diversity index (SHDI) and evenness index (SHEI) increased slowly compared with those in 2020, while the contagion index (CONTAG) and landscape separation index (DIVISION) decreased slightly. The cropland protection scenario effectively suppresses fragmentation and improves the overall connectivity and aggregation of the landscape by expanding cropland patches and reducing patch density. However, the land use diversity has declined, and it is necessary to balance cropland protection and ecological diversity. In view of the situation of cropland protection, it is suggested to carry out comprehensive land consolidation while ensuring the quantity of cropland, which can be improved by means of vegetation restoration and wetland construction, so as to prevent the ecological monotony caused by single cropland and take into account the efficiency of agricultural production and the maintenance of ecological functions.

In the ecological protection scenario in 2030, the patch density (PD) and landscape shape index (LSI) increased slightly. The patch density is the lowest, and the shape index is at a medium level, indicating that the landscape is the most compact and the patch shape is relatively simple. The contagion index (CONTAG) is slightly higher than other scenarios. The patch cohesion index (COHESION) increased significantly, it shows that the landscape aggregation and connectivity reach the highest level. Landscape division index (DIVISION) decreased sharply, significantly lower than other scenarios. Shannon’s diversity index (SHDI) and Shannon’s evenness index (SHEI) remained basically stable, reflect the landscape diversity is still maintained at a high level, but the uniformity is slightly lower. The ecological protection scenario achieved the smallest number of patches, the largest patch area and the highest connectivity, and the most compact landscape structure, which was conducive to the continuity of ecological processes and species migration. The landscape’s ability to resist risks is enhanced, and the landscape security is significantly improved. It is the optimal path of regional land space optimization.

#### Suggestions on the spatial layout of future ecological protection areas

Based on the landscape pattern characteristics under the four scenarios, following the principles of connectivity priority, functional diversity, and human activity compatibility, it is recommended that the following three spatial levels be listed as priority ecological protection areas to achieve ‘protection-development’ interconnection (Table 9).

**Table 9:** Suggestions on the spatial layout of future ecological protection areas.

Regional type	Selection basis	Concrete scope	Concrete measure
Core ecological protection area	*The patch area is the largest and the connectivity is the highest *In the ecological protection scenario, the built-up land of forest, grassland and water is in a large contiguous state *It has important ecological functions	*The continuous primitive forest belt in the mountainous area of northern Guangxi *The main river valleys and their wetland systems on both sides	*The core ecological protection area will be included in the provincial / national nature reserve, the implementation of permanent ban on development *An ecological monitoring station was established to monitor forest coverage, wetland water level and biodiversity index in real time
Ecological corridor buffer zone	*Patch fragmentation is the most serious in urban development scenarios, and connectivity needs to be improved through corridors *Areas with significant increases in patch density (PD) and landscape shape index (LSI)	*The valley corridor connecting the core protection area and the green belt along the river *Cropland-forest ecotone in the periphery of town	*Delineate the red line of the ecological corridor in the rapid urban development area and prohibit large-scale hardening projects *Promote forest belt and wetland restoration projects, using local tree species and wetland plants for vegetation restoration
Cropland ecological composite area	*In the scenario of cropland protection, a large area of cropland has been formed, but the ecological diversity is slightly lower, so it is necessary to add ecological elements inside or on the edge of cropland *Taking into account food security and ecological functions	*Hilly farming area dominated by terraces *Forest network and wetland small patches were set on the edge of cropland	*A small area of forest and wetland is set up inside or on the edge of cultivated land, and farmers’ participation is encouraged through ecological compensation *Guide precision agriculture technology, improve the output rate of cropland, and reduce the demand for new cropland

According to the landscape characteristics of different scenarios, by defining the core ecological protection area, constructing the buffer zone of ecological corridor and promoting the ecological composite model of cropland, with the support of multi-dimensional policies of system, economy and science and technology, the ecological security pattern of northern Guangxi can be improved while ensuring the regional economic and social development, so as to lay a solid foundation for the sustainable development in the future.

## Discussion

Through the combination of multi-source remote sensing, landscape pattern index and scenario simulation, this study systematically explained the spatial and temporal evolution of land use and its driving mechanism in the context of rapid urbanization in northern Guangxi. From 1980 to 2020, the rapid growth of population and economy in northern Guangxi promoted the process of urbanization. A large number of cropland and forest accelerated the transformation to built-up land, and experienced a significant ‘bidirectional succession and increased artificialization’ process of natural landscape shrinkage and artificial landscape expansion. This result is consistent with the research conclusions of the karst area in the Li River Basin, reflecting the general influence model of high-intensity human activities on landscape structure^39^.

At the same time, the landscape pattern in northern Guangxi showed a trend of increasing fragmentation and decreasing connectivity. Landscape fragmentation leads to the obstruction of species migration and energy flow in ecological processes, which in turn weakens the connectivity and resilience of ecosystems. Although the increase in diversity index indicates that land use types are more abundant, this ‘surface diversity’ is difficult to be transformed into actual biodiversity improvement and ecosystem services enhancement if there is a lack of functionally connected ecological networks.

The in-depth analysis of the driving mechanism shows that socio-economic factors are the dominant force in the evolution of landscape pattern in northern Guangxi. Its core mechanism is that population growth and economic development directly drive the demand for urban built-up land and infrastructure construction. This process is often achieved by occupying marginal cropland and forest, resulting in the segmentation and erosion of natural landscape patches and deepening landscape fragmentation. The study of Shennongjia also shows that social and economic factors are the dominant factors leading to the change of regional landscape pattern^40^.

The multi-scenario simulation prediction shows that under the natural development scenario and urban development scenario, urban expansion has the risk of breaking through the ecological protection red line and encroaching on permanent basic cropland, which is closely related to the control requirements of ‘three zones and three lines’ in land and space planning, highlighting the urgency of strictly implementing the ‘three zones and three lines’ boundary and strengthening the rigid constraints of urban development boundary. Under the ecological protection scenario, it can effectively reverse the trend of landscape fragmentation and connectivity decline, and become the optimal path for regional land space optimization by inhibiting disorderly expansion and improving the aggregation degree of ecological land. This is highly consistent with the current China’s advocacy of the concept of ecological civilization and the ‘double evaluation’ requirements of land and space planning^41–43^ (‘Guidelines for the evaluation of resource and environmental carrying capacity and suitability of land and space development (Trial)’, ‘National Land and Space Planning Outline (2021–2035)’). The scenario simulation of this study is in line with the ‘double evaluation’ framework, and the resource carrying capacity and development suitability are evaluated simultaneously, and the priority ecological protection area is defined, which reflects the practical value of ‘double evaluation’ in planning and decision-making.

This study has clear implications for the territorial spatial planning of northern Guangxi. In the future, we should pay attention to the construction of ecological networks, restore forest belts and wetland corridors in key locations, and enhance the spatial connectivity of patches. Rigid control of space, identification of ecological protection red line, permanent basic cropland, urban development boundary, to prevent the built-up land to the ecological red line penetration, to ensure the amount of cropland, rigid implementation of the ‘three zones and three lines’. Optimize the layout of land use, reduce the fragmentation of cropland through land integration, and take into account agricultural production and ecological protection. By strengthening the assessment of ecosystem services, combining quantitative tools such as models, monitoring the specific impact of landscape changes on regulatory services, and providing a solid ecological foundation for regional sustainable development.

The limitations of this study mainly lie in the limitations of data resolution on the characterization of micro-landscape details and the adaptive challenges of the model to the simulation of sudden major policy adjustments. Future research can introduce higher-resolution data and strengthen the dynamic coupling between models and policy scenarios to deepen the understanding of the complex mutual feedback mechanism of the human-land system in karst areas and provide more accurate scientific and technological support for high-quality regional development.

## Conclusions

In this study, ArcGIS Desktop 10.8, Fragstats4.2 software and PLUS model were used to quantitatively analyze the temporal and spatial variation characteristics of land use and landscape pattern in northern Guangxi from 1980 to 2020, and to analyze the evolution trend of land use structure and landscape pattern in northern Guangxi in 2030 under different scenarios. The main conclusions are as follows :

1. From 1980 to 2020, the background landscape advantages of cropland, forest and grassland in northern Guangxi decreased by 307 km^2^, 140 km^2^ and 68 km^2^, respectively. The built-up land showed a rapid growth trend, with an increase of about 444 km^2^. Over the past 40 years, land use changes in the northern Guangxi region have exhibited a landscape structure characterised by ‘bidirectional succession and increased artificialization’.
2. From 1980 to 2020, the landscape pattern in northern Guangxi showed a trend of increasing fragmentation and decreasing connectivity. Land use change has led to significant changes in the landscape pattern in Guangxi, the overall ecological landscape self-regulation ability has been declining, and the instability of the landscape pattern has increased.
3. From 1980 to 2020, the land use change in northern Guangxi was driven by socio-economic and natural environmental factors. Population growth was the dominant driving factor of land use change. Economic activities had a significant impact on cropland, forest, grassland and built-up land. The water area was controlled by topography and hydrology, and the unused land was mainly restricted by the distance of rural settlements.
4. The multi-scenario simulation in 2030 shows that under different scenarios, the degree of landscape fragmentation has increased, the shape of patches has become more complex, and the interference of human activities has increased. Under the natural development and urban development scenarios, the fragmentation is particularly significant and the ecological connectivity is reduced; although the cropland protection scenario inhibits fragmentation, it may sacrifice ecological diversity; the ecological protection scenario achieves the most compact landscape structure, the highest connectivity and aggregation, significantly improves the landscape security and ecological process continuity, and is the optimal path for regional sustainable development. It is recommended to give priority to ecological protection scenarios and pay attention to the balance of ecological functions in cropland protection scenarios.

## Supplementary Information

Below is the link to the electronic supplementary material.

Supplementary Material 1

## Data Availability

National Platform for Common Geospatial Information Services: https://www.tianditu.gov.cn/(accessed on 20 November 2024) (see Supplementary Materials). Geospatial Data Cloud: https://www.gscloud.cn/Resource and Environmental Science Data Platform: https://www.resdc.cn. National Catalogue Service For Geographic Information: https://www.webmap.cn/main.do?method=index.

## References

[1] Turner, M. G. Landscape ecology: What is the state of the science?. *Annu. Rev. Ecol. Evol. Syst.* **36**, 319–344. 10.1146/annurev.ecolsys.36.102003.152614 (2005).

[2] Jiang, H. et al. Spatiotemporal evolution and driving factors of ecosystem service bundle based on multi-scenario simulation in Beibu Gulf urban agglomeration. *China. Environ. Monit. Assess.* **196**, 22. 10.1007/s10661-024-12663-6 (2024). 10.1007/s10661-024-12663-6 38735886

[3] Pearson, D., Martínez-López, J., Rescia, A. J., Baldwin, R. & Pastur, G. J. M. Feature papers in landscape ecology: An editorial overview. *Land* **13**, 342. 10.3390/land13030342 (2024).

[4] Wang, X. et al. Urban ecosystem: Highly spatial heterogeneity. *Acta. Ecol. Sin.* **40**, 5103–5112. 10.5846/stxb201901300222 (2020).

[5] Turner, M. G. Landscape ecology: The effect of pattern on process. *Annu. Rev. Ecol. Evol. Syst.* **20**, 171–197. 10.1146/annurev.ecolsys.20.1.171 (1989).

[6] Gardner, R. H. & O’Neill, R. V. Pattern, process, and predictability: the use of neutral models for landscape analysis. *Ecol. Stud.* **82**, 289–307 (1991).

[7] Forman, R. T. T. *Land mosaics: The ecology of the landscapes and regions*. (Cambridge university press, 1995).

[8] Wu, J. Landscape sustainability science: Ecosystem services and human well-being in changing landscapes. *Landscape Ecol.* **28**, 999–1023. 10.1007/s10980-013-9894-9 (2013).

[9] Wu, J. et al. Landscape sustainability science and the sustainable development goals. *Geogr. Sustain.*10.1016/j.geosus.2025.100309 (2025).

[10] Wang, J., Zhao, W., Ding, J. & Liu, Y. Shifting research paradigms in landscape ecology: Insights from bibliometric analysis. *Landscape Ecol.*10.1007/s10980-025-02082-4 (2025).

[11] Kowe, P., Mutanga, O. & Dube, T. Advancements in the remote sensing of landscape pattern of urban green spaces and vegetation fragmentation. *Int. J. Remote Sens.* **42**, 3797–3832. 10.1080/01431161.2021.1881185 (2021).

[12] Pătru-Stupariu, I. & Nita, A. Impacts of the European landscape convention on interdisciplinary and transdisciplinary research. *Landscape Ecol.* **37**, 1211–1225. 10.1007/s10980-021-01390-9 (2022).

[13] Zhou, H. C., Sun, Z. L. & Xie, Y. C. *Research on geographic cellular automata*. (Science Press, 1999).

[14] Yang, G., Liu, Y. & Wu, Z. Analysis and simulation of land-use temporal and spatial pattern based on CA-Markov model. *Geomatics Inform. Sci. Wuhan Univ.* **32**, 414–418. 10.3969/j.issn.1671-8860.2007.05.010 (2007).

[15] Zhang, Y., Zhao, S. & Verburg, P. H. CLUE-S and its application for simulating temporal and spatialchange of land use in Naiman Banner. *J. Nat. Res.* **18**, 310–318 (2003).

[16] Wang, B. et al. The weight of neighborhood setting of the FLUS model based on a historical scenario: A case study of land use simulation of urban agglomeration of the Golden Triangle of Southern Fujian in 2030. *Acta Ecol. Sin.* **39**, 4284–4298. 10.5846/stxb201808021649 (2019).

[17] Liang, X. et al. Understanding the drivers of sustainable land expansion using a patch-generating land use simulation (PLUS) model: A case study in Wuhan. *China. Comput. Environ. Urban Syst.* **85**, 14. 10.1016/j.compenvurbsys.2020.101569 (2021).

[18] Liu, J. et al. Prediction of land use for the next 30 years using the PLUS model’s multi-scenario simulation in Guizhou Province. *China. Sci. Rep.* **14**, 13143. 10.1038/s41598-024-64014-7 (2024). 38849508 10.1038/s41598-024-64014-7 PMC11161487

[19] Lin, G. et al. Morphological characteristics and spatial evolution laws of rural settlements at peak-cluster depressions in rocky desertification areas. *J. Soil Water Conserv.* **39**, 348–358 (2025).

[20] Li, H., Niu, X., Wang, B. & Zhao, Z. Coupled coordination of ecosystem serices and landscape patems: Take the grain for green project in the Wuling mountain area as an example. *Acta Ecol. Sin.* **40**, 4316–4326. 10.5846/stxb201911152443 (2020).

[21] Zhou, B., Chen, G., Zhao, J., Yin, Y. & Yu, Z. Spatiotemporal evolution and trade-offs and synergies of ecosystem services in the karst region of southeast Yunnan. *Water Res Hydropower Eng.* **55**, 123–142 (2024).

[22] Lai, G., Hu, B., Li, M., Lin, S. & Deng, Y. Dynamic changes of ecological-living-production land and geographical detect of their driving forces in Southwest Guangxi-Beibu Gulf Zone. *Res. Soil Water Conserv.* **28**, 236–243 (2021).

[23] Ni, C., He, W. & Yao, Y. Assessment of cultivated land use change in Guilin using the GeoSOS-FLUS model. *Bullet. Surv. Map.* **5**, 35–40 (2024).

[24] Wei, Q., He, W., Wang, J., Zhou, X. & Yao, Y. Spatial and temporal evolutionary characteristics of landscape pattern of a typical karst watershed based on GEE platform. *J. Res. Ecol.* **14**, 928–939. 10.5814/j.issn.1674-764x.2023.005.004 (2023).

[25] Sun, R., Sun, L., Su, X. & Chen, L. A review on the coupling research of landscape patterns and ecological processes: Consistency and innovation. *Acta Ecol. Sin.* **41**, 415–421. 10.5846/stxb201912102684 (2021).

[26] Yang, J. et al. A review on the driving factors and their characteristics in urban landscape pattern changes. *Acta. Ecol. Sin.* **44**, 10486–10498 (2024).

[27] Li, H., He, W., Wang, J., Yang, S. & Yao, Y. Multi-scenario prediction of ecosystem service values based on the PLSR-FLUS-MarKov model: A case study of Lijiang River basin. *J. Hydroecol.* **46**, 203–212 (2025).

[28] Qi, T., Wang, Y. & Wang, W. A review on visual landscape study in foreign countries. *Prog. Geogr..* **32**, 975–983 (2013).

[29] Han, H. et al. The land-use and land-cover change characteristies and driving forces of cultivated land in Central Asian countries fom 1992 to 2015. *Chin. J. Eco-Agric.* **29**, 325–339 (2021).

[30] Zhang, Z. et al. Dynamie variation of landseape pattern of land use in Songyuan city in nearly 20 years. *Chin. Agric. Sci. Bullet.* **30**, 222–226 (2014).

[31] Cheng, J., Cheng, J., Wu, J. & Xu, Y. Changes of land use and ecosystem service functions in Yangtze River basin from 2000 to 2010. *Res. Environ. Yangtze Basin.* **26**, 894–901 (2017).

[32] Li, J. & Ma, L. Coupling relationship between land use changes and its eco-environmental effect in Yanchi county. *Sci. Soil Water Conserv.* **18**, 19–25 (2020).

[33] He, Q., Xie, D., Wang, S., Yan, J. & Chen, L. Land use transformation and its eco-environmental effects in Beibei district, Chongqing. *Res. Soil Water Conserv.* **26**, 290–296 (2019).

[34] Luo, Y., Liu, W., Qi, A., Qu, Y. & Liu, W. Spatiotemporal evolution of land use and multi-scenario simulation of ecological space in Dawenhe River Basin. *Water Sav. Irrig.* **8**, 44–52 (2024).

[35] Wang, B. *Spatial-temporal pattern evolution andmulti-scenario simulation of land use conflict in Poyang Lake Area based on PLUS model.* Master thesis, Jiangxi Normal University, (2023).

[36] Gao, L. et al. Multi-scenario simulation and ecological risk analysis of land use based on the PLUS model: A case study of Nanjing. *Sustain. Cities Soc.* **85**, 104055. 10.1016/j.scs.2022.104055 (2022).

[37] Nie, W. et al. Simulating future land use by coupling ecological security patterns and multiple scenarios. *Sci. Total Environ.* **859**, 160262. 10.1016/j.scitotenv.2022.160262 (2023). 36400298 10.1016/j.scitotenv.2022.160262

[38] Xiao, D. & Zhong, L. Ecological principles of landscape classification and assessment. *Chin. J. Appl. Ecol.* **9**, 217–221 (1998).

[39] Fan, Q., Ma, J., Yu, M. & He, G. Comprehensive disturbance evaluation of karst and its spatial characteristics in the Lijiang River Basin, Guilin China. *Acta Ecol. Sin.* **44**, 1404–1417 (2024).

[40] Cao, J., Deng, Z., Hu, Y. & Wu, Y. Spatial and temporal evolution and driving forces of the landscape pattern in Shennongjia Forestry District. *J. Zhejiang A&F Univ.* **38**, 155–164 (2021).

[41] Zhao, Y. et al. Distinguishing the effects of land use policies on ecosystem services and their trade-offs based on multi-scenario simulations. *Appl. Geogr.* **151**, 102864. 10.1016/j.apgeog.2022.102864 (2023).

[42] Yu, M., Liu, Y. & Zhang, Y. Multi-scenario prediction of future land use change and landscape ecological risk on the Qingzang Plateau. *Res. Environ. Yangtze Basin.* **33**, 2204–2218 (2024).

[43] Zhong, Z., Zhang, H., Hong, L., Liu, G. & Luo, W. Territorial space baseline control from the perspective of ecological civilization: “Double evaluation” and monitoring-evaluation-warning. *J. Nat. Res.* **35**, 2415–2427 (2020).

